# RNA methylation in plants: An overview

**DOI:** 10.3389/fpls.2023.1132959

**Published:** 2023-03-01

**Authors:** Harshraj Shinde, Ambika Dudhate, Ulhas S. Kadam, Jong Chan Hong

**Affiliations:** ^1^ Department of Animal and Food Sciences, College of Agriculture, Food and Environment, University of Kentucky, Lexington, KY, United States; ^2^ Sequencing and Genome Discovery Center, Stowers Institute for Medical Research, Kansas City, MO, United States; ^3^ Plant Molecular Biology and Biotechnology Research Center (PMBBRC), Division of Life Science and Division of Applied Life Science (BK21 Four), Gyeongsang National University, Jinju-daero, Jinju, Gyeongnam, Republic of Korea; ^4^ Division of Plant Sciences, University of Missouri, Columbia, MO, United States

**Keywords:** RNA methylations, plant development, writers, gene regulation, software

## Abstract

RNA methylation is an important post-transcriptional modification that influences gene regulation. Over 200 different types of RNA modifications have been identified in plants. In animals, the mystery of RNA methylation has been revealed, and its biological role and applications have become increasingly clear. However, RNA methylation in plants is still poorly understood. Recently, plant science research on RNA methylation has advanced rapidly, and it has become clear that RNA methylation plays a critical role in plant development. This review summarizes current knowledge on RNA methylation in plant development. Plant writers, erasers, and readers are highlighted, as well as the occurrence, methods, and software development in RNA methylation is summarized. The most common and abundant RNA methylation in plants is N6-methyladenosine (m^6^A). In Arabidopsis, mutations in writers, erasers, and RNA methylation readers have affected the plant’s phenotype. It has also been demonstrated that methylated TRANSLATIONALLY CONTROLLED TUMOR PROTEIN 1-messenger RNA moves from shoot to root while unmethylated TCTP1-mRNA does not. Methylated RNA immunoprecipitation, in conjunction with next-generation sequencing, has been a watershed moment in plant RNA methylation research. This method has been used successfully in rice, Arabidopsis, Brassica, and maize to study transcriptome-wide RNA methylation. Various software or tools have been used to detect methylated RNAs at the whole transcriptome level; the majority are model-based analysis tools (for example, MACS2). Finally, the limitations and future prospects of methylation of RNA research have been documented.

## 1 Introduction

In epigenetics, DNA and histone methylations are critical aspects of genetic regulation. In the past few decades, rapid progress in understanding DNA and histone methylation has been achieved (Greer and Shi, 2012). DNA and histone methylation control different aspects of plants’ development and adaptation. Both these processes are also involved in silencing repetitive elements, which helps maintain genomic stability (Liu et al., 2010; Bartels et al., 2018). In recent years, RNA methylation has emerged as an essential regulatory mechanism in plant epigenetics (Hu et al., 2019a). However, the mechanism of RNA methylation is poorly explored in plants compared to animals. The machinery involved in methylation (writing), demethylation (erasing), and reading are well-documented in animals (Yang et al., 2018). However, the homologs of these writers, erasers, and readers are not thoroughly studied in plants(Hu et al., 2019a). In Arabidopsis, mutations in writers, erasers, and readers of RNA methylation have impacted phenotypic characteristics, showing the importance of RNA methylation in plant growth and development. Transcriptome-wide study on RNA methylation is known as epitranscriptomics. Only a handful of studies are performed on epitranscriptome analysis of plants. Novel RNA methylation marks such as m^3^C were identified in Arabidopsis using the epitranscriptome approach. The presence of an m^3^C methylation mark is linked with transcripts’ instability and high turnover rates (Vandivier et al., 2015). Epitranscriptome analysis in callus and leaf tissues of rice reveals the yields of 8,138 and 14,253 m^6^A-modified genes, respectively. Transcription termination and transcription initiation sites exhibited the presence of most of the m^6^A -modified nucleotides (Li et al., 2014). This study in rice reveals the role of m^6^A in gene silencing and activation.

Although RNA methylation plays an essential role in plants, understanding the role of RNA methylation in plants is just beginning. This review has documented a brief overview of current research on plant RNA methylation. Different types of methylation are added to RNA by different methyltransferases and have been studied to various extents. Our review primarily focuses on m^6^A methylation and the machinery involved in its regulation. Here, we discuss current findings, available methods to study RNA methylation in plants, valuable tools to analyze plant epitranscriptome data, limitations, and future prospectus.

## 2 Writers, erasers, and readers of RNA methylation in plants

RNA methylating enzymes are often considered as writers, whereas readers act as effectors by binding to the methylated nucleotide, and erasers remove the methylation to reset the effect (Lim and Pawson, 2010). In 1994, genes encoding m^6^A writer, methyltransferase-like 3 (METTL3), and METTL14 were recognized in animals (Bokar et al., 1994). Later in 2008 and 2017, the orthologs of METTL3, MTA, and METTL14, MTB, were identified in Arabidopsis (Zhong et al., 2008; Růžička et al., 2017). In Arabidopsis, methyltransferase A/B (MTA and MTB) proteins are involved in embryo development (Hu et al., 2019a). Few other writer proteins like FIP37 (FKBP12 Interacting protein 37), VIR (Virilizer), and HAKAI/CBLL1 (Casitas B-lineage lymphoma-transforming sequence-like protein 1) were also reported in plants (Arribas-Hernández and Brodersen, 2020). In Arabidopsis, FIP37 mutants exhibit massive over-proliferation of stem apical meristem without aerial organs (Shen et al., 2016).

RNA methylation erasers or demethylases are responsible for converting methylated nucleotide to normal. Erasers should be critical to study the role of RNA methylation in plants. In eukaryotes, the erasing of methylation marks are achieved by α-ketoglutarate-dependent dioxygenase (AlkB) homolog (ALKBH) proteins (Zaccara et al., 2019). ALKBH erases alkyl and methyl groups from DNAs, RNAs, and proteins. Interestingly, some ALKBH family members, like ALKBH8, contain both methyltransferase and demethylase activities in animals (Pastore et al., 2012). In Arabidopsis, *AtALKBH9B* acts as an eraser that removes m^6^A marks from the Arabidopsis and alfalfa mosaic virus (AMV) RNAs during infection to generate host resistance. Inhibition of *AtALKBH9B* increased the relative abundance of m^6^A marks on AMV RNAs, impairing the systemic invasion of the plant (Martínez-Pérez et al., 2017). Another eraser protein ALKBH10B is noticed to be involved in flowering and vegetative growth maintenance. In Arabidopsis, the *alkbh10b* mutant delays flowering and vegetative growth repression (Duan et al., 2017).

**Figure 1:**
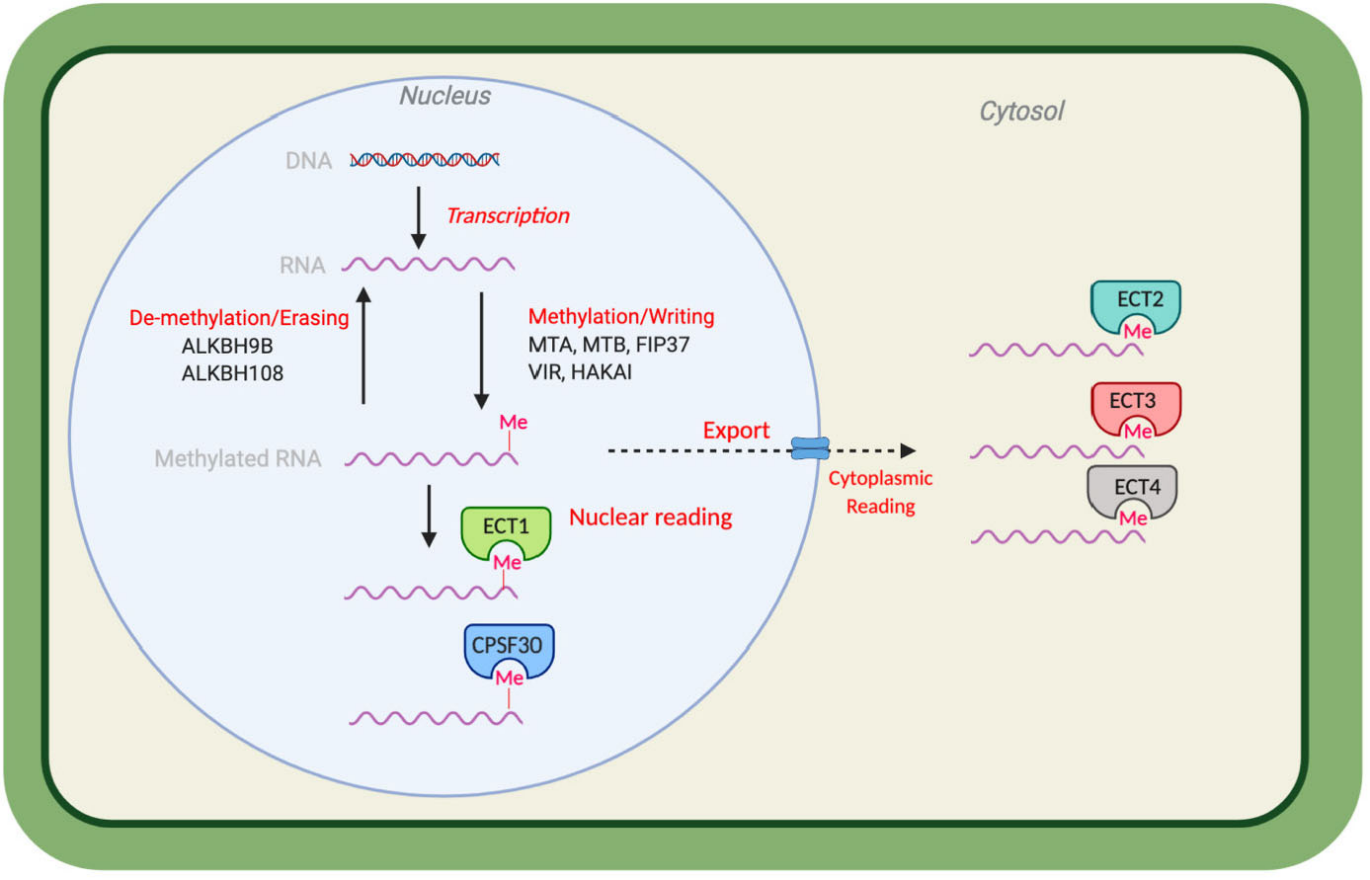
The process of RNA methylation in plants. Methylation (writing), demethylation (erasing), and reading of RNA methylation along with their respective proteins.

A mechanism by which methylation affects the expression of RNA is by recruiting methylation loci binding proteins (readers) (Zaccara et al., 2019). In Plants, ECT (EVOLUTIONARILY CONSERVED C-TERMINAL REGION) proteins are methylation loci binding proteins. Among plant nuclear readers, ECT1 and CPSF30 (Cleavage and Polyadenylation Specificity Factor 30) are involved in calcium signaling and abnormal transcription termination, respectively. Among cytoplasmic readers, Arabidopsis ECT2 targets many m^6^A-containing mRNAs, including TTG1 (TRANSPARENT TESTA GLABRA1), ITB1 (IRREGULARTRICHOMEBRANCH1), and DIS2 (DISTORTED TRICHOME2), which are involved in trichome development. It has also been studied that ECT2 is responsible for mRNA stability (Wei et al., 2018). A detailed illustration of plant writers, readers, and erasers is given in **Figure 1**. In Arabidopsis, two methyltransferase enzymes, TRM4A and TRM4B are responsible for writing the m^5^C methylation. They are orthologs of human m^5^C methyltransferase. The loss of function mutation in the *TRM4A* gene does not modify any visible phenotypes. However, the loss of *TRM4B* reduces root length, implying its role in root growth (David et al., 2017; Hu et al., 2019a). However, erasers and readers of m^5^C are not currently being studied in plants.

Little is known about writers, erasers, and readers in plants; therefore, more future studies are required to elucidate the in-detail role of these proteins in plants.

## 3 Function of RNA methylation in plant development

In plants, m^6^A (6-methyladenosine) methylation was first reported in 1979 in maize, oat, and wheat (Nichols, 1980; Nucleosides and Chromatography, 1980). Till now, m^6^A has been the most studied RNA methylation in plants. Besides m^6^A, m^5^C (5-methylcytosine), pseudouridine, and C-U editing of mitochondrial and chloroplast mRNA are commonly studied in RNA methylation (Arribas-Hernández and Brodersen, 2020).

mRNAs move to distant body parts to potentially act as signaling. A study conducted in *Arabidopsis* using the meRIP-Seq approach shows that m^5^C modification of mobile RNA modification plays a crucial role in facilitating their transport and presents evidence that mobile m^5^C-modified *TCTP1* (TRANSLATIONALLY CONTROLLED TUMOR PROTEIN 1) is translated in target cells and changes root growth (Yang et al., 2019a). In maize, m^6^A methylation shows correlations with the translational status (Luo et al., 2020). In Seagrass, global m^6^A RNA methylation widely contributes to circadian regulation and potentially affects their photo-biological behavior (Ruocco et al., 2020). Additionally, a report on m^6^A methylation in Arabidopsis showed that affects microRNA (miRNA) biogenesis, which demonstrates that m^6^A methylation is necessary to maintain levels of mature miRNAs and their precursors (Bhat et al., 2020). A study on rice has discovered that m^6^A methylation is involved in the pathogenicity of the rice blast fungus *Pyricularia oryzae* (Shi et al., 2019). Another study in maize discovered that m^6^A methylation is responsible for early-stage callus induction; in this study, genes involved in callus induction, i.e., *BABY BOOM* and *LBD*, underwent m^6^A methylation, increasing their expression and promoting callus induction (Du et al., 2020).

Moreover, the fruit of tomatoes showed a molecular link between DNA methylation and RNA methylation (m^6^A) during fruit ripening. In fruits of tomato, the ripening-deficient *Colorless non-ripening* (*Cnr*) mutant, which harbors DNA hypermethylation, approximately 1100 transcripts display increased m^6^A levels. Further analysis confirmed that the increase in m^6^A methylation in *Cnr* mutant fruit is associated with the decreased expression of RNA demethylase gene *ALKBH2* (Zhou et al., 2019). A recent study in Arabidopsis shows that m^6^A RNA methylation in flowers and is negatively correlated with gene expression, which limits the activation of heat stress-related genes and compromises fertility during heat (Wang et al., 2022).

Epitranscriptome analysis along with high-throughput annotation of modified ribonucleotides pipeline to identify and classify RNA methylation has predicted different methylation such as 3-methyl cytosine (m^3^C), 1-methyl guanosine (m^1^G), and 1-methyl adenosine (m^1^A) in plants. The roles of these marks in photosynthesis, response to cold, osmotic stress, etc., have been predicted using gene ontology analysis (Vandivier et al., 2015; Liang et al., 2020). RNA ribose methylation sequencing (RiboMeth-Seq) is used to detect methylation marks in ribosomal RNAs. A study in Arabidopsis where RiboMeth-seq was used to profile ribosomal RNAs identified 111 cytoplasmic rRNA marks. These identified marks will help study rRNA methylation’s role in plants (Wu et al., 2021a).

The organelle-associated RNAs are highly m^6^A-methylated (98−100% of transcripts in chloroplasts and 86−90% in mitochondria). Around 4-6 m^6^A sites per transcript were identified in both organelles. These methylation marks directly influence gene expression, as the negative correlation between m^6^A methylation and gene expression has been observed in both organelles. The high levels of RNA methylation in chloroplast and mitochondrial RNAs suggest the role of RNA methylation in organelles’ functioning. Considering the importance of chloroplast and mitochondria in photosynthesis and energy metabolism, understanding the role of RNA methylation in these organelles will answer many unsolved questions (Manduzio and Kang, 2021).

A recent study in *Arabidopsis thaliana* characterized the biological roles of various m^6^A writers (Wong et al., 2023). This study identified the role of m^6^A writers in biological processes such as photosynthesis, stress/defense response, cell growth, metabolic processes, and so on. The research studies explained above are summarized in **Table 1**.

**Table 1:** Recent studies on RNA methylation in plants.

	Key findings of study	Approach	Plant	Reference
1	RNA methylation contributes to mRNA mobility and root growth	m^5^C antibody mediated immunoblot and MeRIP-Seq	*Arabidopsis thaliana*	(Yang et al., 2019a)
2	Relation of m^6^A methylation with translation status	Transcriptome wide m^6^A profiling and polysome analysis	Maize	(Luo et al., 2020)
3	m^6^A contributes to circadian regulation & photobiological behavior	Global m^6^A quantification by ELISA	Seagrass	(Ruocco et al., 2020)
4	m^6^A methylation affects microRNA biogenesis.	mRNA adenosine methylase (MTA) mutant studies and small RNA sequencing	*Arabidopsis thaliana*	(Bhat et al., 2020)
5	m^6^A methylation is involved in asexual reproduction and pathogenicity of rice blast fungus.	Gene knock-out of rice blast fungus & m^6^A RNA methylation quantification assay	Rice	(Shi et al., 2019)
6	m^6^A methylation is responsible for early-stage callus induction.	Epitranscriptome analysis and RNA sequencing	Maize	(Du et al., 2020)
7	Role of RNA methylation in tomato fruit ripening	Epimutant analysis and Epitranscriptome analysis	Tomato	(Zhou et al., 2019)

The epitranscriptome engineering is a promising tool for achieving food security by editing RNA methylation sites (m^6^A, m^5^C, m^1^A, m^3^C, and so on) through various genome-editing technologies (Shoaib et al., 2022). Furthermore, alternative splicing in plants provides an additional regulatory mechanism and plays key role in plant development (Kadam et al., 2014a; Kadam et al., 2014b; Kadam et al., 2017) nevertheless, impact of RNA methylation on alternatively spliced variants is unknown. In the future, RNA methylation will be a potential target for crop improvement.

### 3.1 Case studies in plants

#### 3.1.1 RNA methylation promotes mRNA transport

**Figure 2:**
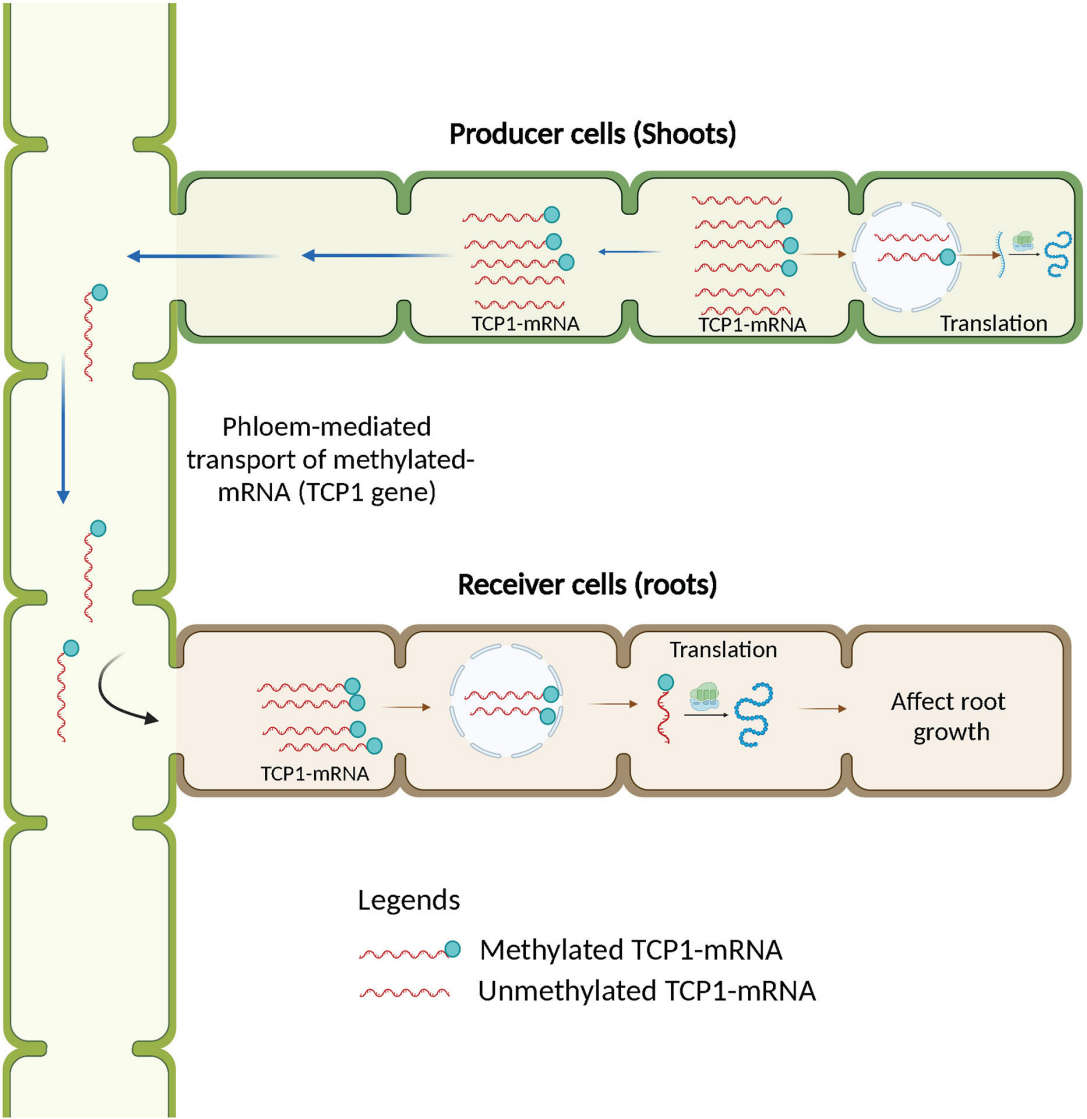
Schematic diagram depicting role of methylation in transporting TCTP1-mRNA (TRANSLATIONALLY CONTROLLED TUMOR PROTEIN 1-messenger RNA) from shoots to roots.

As the main transport route, the vascular system of plants, including the xylem and phloem, plays a significant role in the growth and development process (Lucas et al., 2013). Phytohormones, sugars, proteins, water, and RNA molecules are transported from source to sink (Lemoine et al., 2013). RNAs act as signaling upon transportation. In plants, RNA transport mechanisms are mainly related to RNA motifs, including the polypyrimidine sequence, the RNA-related transfer sequence, the single nucleotide mutation, the tRNA-type structure, and the nonreporting region (Wang et al., 2021a). Along with this, methylation of RNA also contributes to the transport/movement of RNAs in plants. A study using the model plant *Arabidopsis thaliana* by Yang et al. first time provided evidence that RNA methylation is involved in the transport of plant mRNA (Yang et al., 2019a). This study pointed out that cytosine methylation is required for the mobility of TCTP1-mRNA (TRANSLATIONALLY CONTROLLED TUMOR PROTEIN 1-messenger RNA). These methylated TCTP1 mRNA moved from shoot to root *via* phloem and affected root growth in *Arabidopsis thaliana.* (**Figure 2**). Based on methylated RNA Immunoprecipitation sequencing study, the database with the name “Cucume” (http://cucume.cn/) has been developed for cucumber (*Cucumis sativus* L.) and pumpkin (*Cucurbita moschata*). The Cucume database contains information about the m^5^C and m^6^A sites of different tissues and the vascular exudates. The Cucume database also includes graft-transmissible mRNAs identified in previous studies using heterografts. This database will help in understanding of the role of cucurbit RNA methylation in RNA mobility (Li et al., 2023).

#### 3.1.2 Ribosomal RNA methylation is crucial for chloroplast functioning and ABA response

**Figure 3:**
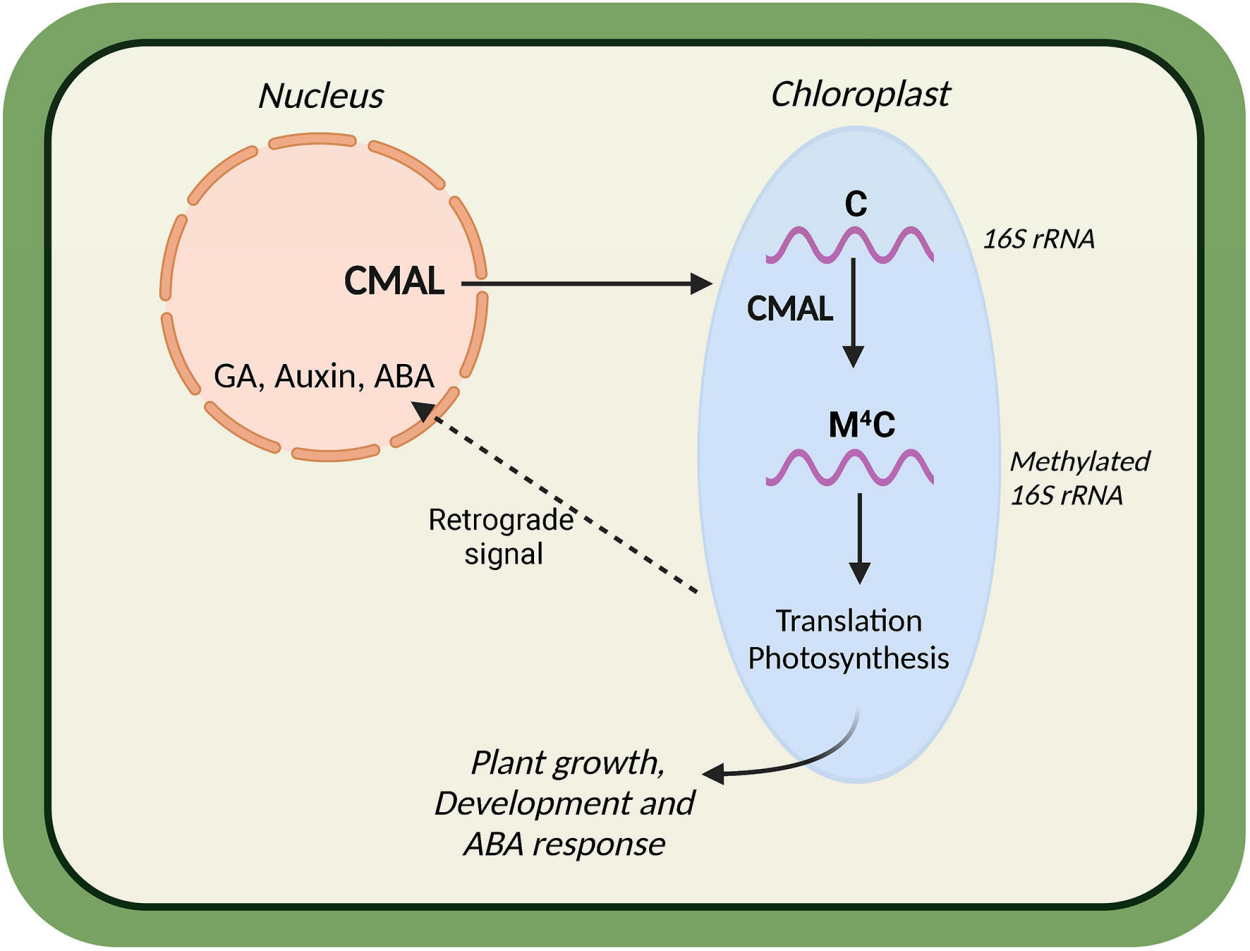
Schematic diagram depicting the role of CMAL (Chloroplast mraW‐Like) in chloroplast development, photosynthesis, and abscisic acid response.

The role of messenger RNA methylation is well recognized (Manduzio and Kang, 2021). However, the nature of ribosomal RNA (rRNA) methylation remains largely unknown. A study in the model plant *Arabidopsis thaliana* revealed that CMAL (Chloroplast mraW‐Like) methyltransferase is responsible for inducing N4‐methylcytidine (m^4^C) type methylation in 16S chloroplast rRNA. The CMAL mutant showed reduced chloroplast biogenesis, altered photosynthetic activity, and stunted growth. The CMAL‐ overexpression lines grew better than the wild type in the presence of abscisic acid (ABA). **Figure 3** shows how the nucleus-encoded CMAL protein is transported into the chloroplast and is responsible for m^4^C methylation in 16S chloroplast ribosomal RNA (**Figure 3**). This methylation is crucial for chloroplast biogenesis and photosynthesis.

Along with this, chloroplast‐to‐nucleus signal affects the expression of plant hormone (GA, auxin, and ABA) signaling-related genes. This study indicates that all these CMAL-mediated processes are essential for plant development and hormone signaling (Tieu Ngoc et al., 2021). Another study (Zou et al., 2020) in Arabidopsis showed that CMAL is involved in plant development by modulating auxin signaling pathways, in which authors uncovered the role of CMAL in ribosome biogenesis (Zou et al., 2020).

## 4 Occurrence of RNA methylation motifs in plant RNAs

An earlier study on maize in 1980 discovered that most of the m^6^A loci are present in the poly-A tail of mRNAs and mainly occur in the R(m^6^A)C (R=A/G) sequence pattern (Nichols, 1980). Based on this study, most of the researchers predicted the role of m^6^A methylation in RNA stability. Later in 2014, a study on *Arabidopsis* found RR(m^6^A) CH (H=A/C/U) as an extended consensus motif. This study also reveals that m^6^A in *Arabidopsis* is enriched around the stop codon, within 3′ untranslated regions (3′ UTRs), and around the start codon (Young Hee Choi, 2014). Nanopore direct RNA sequencing of Arabidopsis shows that loss of m^6^A from 3’UTRs is associated with decreased in transcripts accumulation and defective RNA 3′ end formation (Parker et al., 2019). In addition to RRACH motif, UGUAMM and RAGRAG (R=A/G, H=A/C/U, W=A/U, M=A/C) were located as m^6^A motifs in rice (Li et al., 2014). Epitranscriptome analysis suggested the GGAU and URUAY as plant-specific motifs of m^6^A, as they were reported in plants (Anderson et al., 2018; Wei et al., 2018). A methyltransferase enzyme, TRM4, methylates TLS (tRNA-like structure) motifs on specific tRNAs, as a result of methylation, enhances the stability of tRNAs and avoids degradation caused by environmental changes. TLS was also significantly enriched in mobile mRNA datasets of *Arabidopsis* (Wang et al., 2021a).

However, our knowledge about the occurrence of RNA methylation motifs is incomplete. More single-nucleotide resolution studies are needed to find out motifs enriched for RNA methylation sites.

## 5 Methods of studying RNA methylation in plants

The methods for studying RNA methylation have been divided into two main parts. 5.1. Sequencing-based methods, where data for sequencing is generated and analyzed. 5.2. Non-sequencing-based methods, where sequencing is not generated.

### 5.1 Sequencing-based methods.

#### 5.1.1 Bisulfite conversion

Bisulfite conversion is a chemical method where unmethylated cytosines are deaminated to uracil while methylated cytosine (m^5^C) is left intact. This method is widely used in the research of DNA methylation (Suzuki and Bird, 2008). One limitation of this method in RNA methylation studies is that a large amount of RNA is required as a starting material. Because RNA is incubated at high temperatures in a buffer containing sodium bisulfite, RNA is likely to be degraded. This method is performed along with epitranscriptome studies or RT-PCR-based studies. First total RNA is incubated with sodium bisulfite. Then, bisulfite-converted RNAs are converted to cDNA using primers (stem-loop, oligo-dT or random). After PCR amplification, it is directly used for sequencing (Schaefer et al., 2009). The main disadvantage of this method is most common RNA methylation is m^6^A rather than m^5^C, and lengthy protocol, as compared to other methods.

#### 5.1.2 MeRIP-Seq (Methylated RNA immunoprecipitation sequencing)

**Figure 4:**
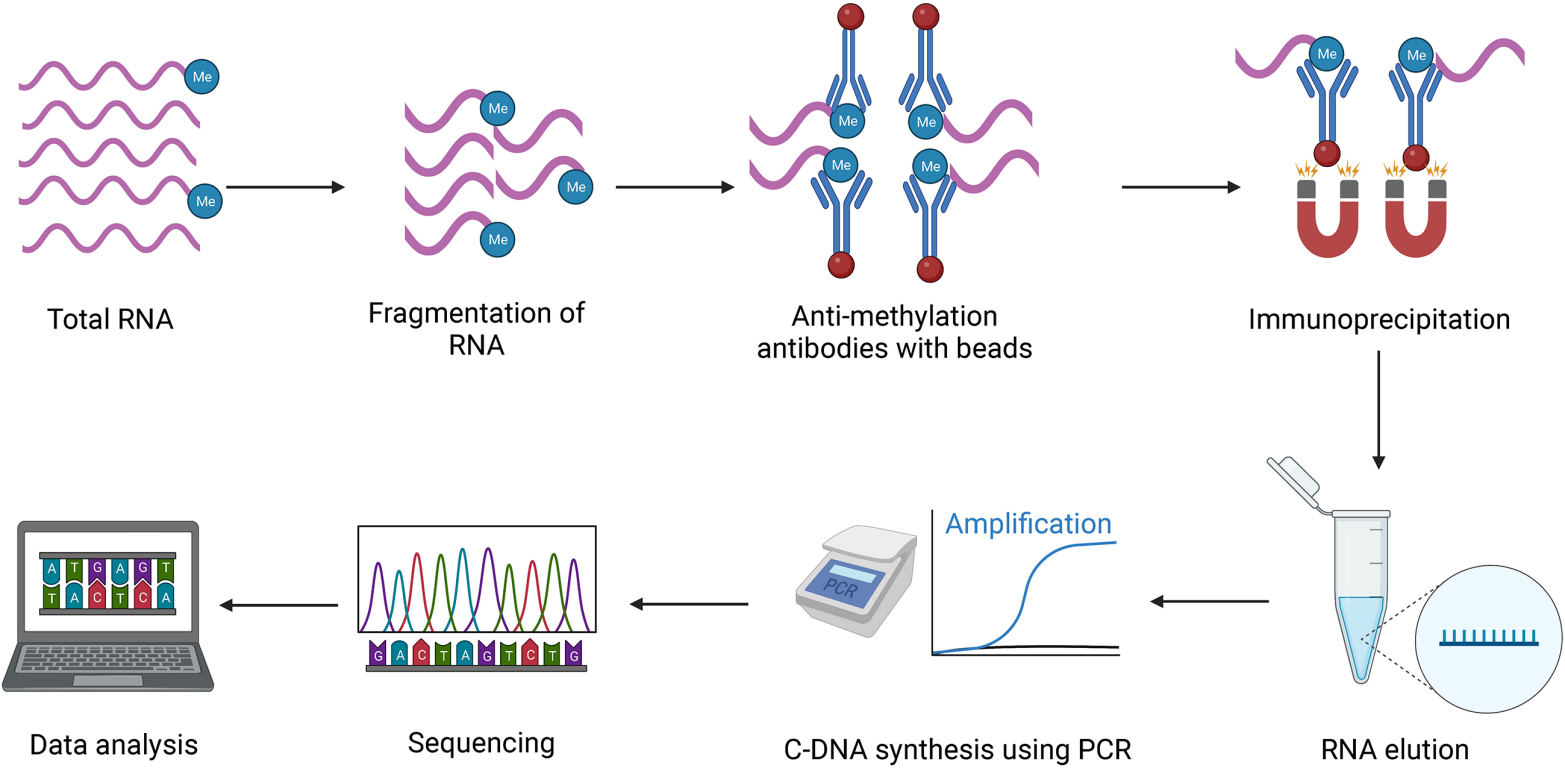
Schematic diagram of meRIP-Seq, 1) Starting RNA 2) Fragmentation of RNA 3) RNA Methylation detection using antibody 4) Immunoprecipitation 5) RNA elution 6) C-DNA synthesis using PCR 7) Sequencing 8) Data analysis.

The development of MeRIP-Seq in 2012 was a milestone in the field of epitranscriptome (Meyer et al., 2012). MeRIP-Seq is most used method to study RNA methylation at the transcriptome level. Meyer et al. and team invented this method to detect the m^6^A level at the transcriptome level (Meyer et al., 2012). This method involves fragmentation of total RNA, binding specific antibodies (e.g., anti-m^6^A and anti-m^5^C), immunoprecipitation of antibody-bound methylated RNA, elution of RNA, cDNA synthesis, and finally, sequencing and data analysis. The whole procedure of meRIP-Seq is given in **Figure 4**. This method has been successfully used in transcriptome-wide detection of RNA methylation in various plants, like *Arabidopsis thaliana*, Brassica, rice, maize and so on (Cui et al., 2017; Hu et al., 2019a; Liu et al., 2020). Limitations of this method are low resolution and requiring a high amount of RNA. The rate of false positive generation is high, as in some cases, antibody exhibits non-specific binding (Mongan et al., 2019). 2’-O-Methylation coupled with MeRIP-Seq comprehensively detect 2’-O-RNA methylation at single-base resolution in different RNAs such as tRNA, mRNA, rRNA, lncRNA, miRNA, *etc*. (Dai et al., 2017).

#### 5.1.3 miCLIP (m^6^A individual-nucleotide resolution cross-linking and immunoprecipitation)

In 2015, miCLIP was developed by Linder et al. to address the overcome drawbacks associated with meRIP-Seq (Linder et al., 2015). This method is useful to study only m^6^A type of RNA methylation. In plants, m^6^A methylation is mainly concentrated in meristems and reproductive organs (Zheng et al., 2020). Hence, in plants, the miCLIP method is primarily used for actively dividing cells, but is also suitable for non-dividing cell. In this method, total RNA containing m^6^A methylation is first fragmented and incubated with an anti- m^6^A antibody. After UV cross-linking, the antibody-RNA complexes are immunoprecipitated using protein A/G affinity beads. Then 3` adapters are ligated to the RNA. Antibody-RNA complexes are then purified by nitrocellulose membrane and eluted using proteinase K, allowing only a small peptide fragment cross-linked at the m^6^A or m^6^A site. RNA fragments are reverse transcribed, which results in mutations or truncations at the cross-link site in the resulting cDNA. Finally, the cDNA is synthesized by PCR and sequenced (Linder et al., 2015; Hawley and Jaffrey, 2019).

#### 5.1.4 Metabolic propargylation for methylation sequencing (MePMe-seq)

Metabolic propargylation for methylation sequencing (MePMe-seq) method has been reported as antibody free RNA methylation detection method. In this method, metabolic labeling with propargyl-selenohomocysteine in along with click chemistry is used to detect N6A and m5C sites in mRNA with single nucleotide precision in the same sequencing run (MePMe-seq). MePMe-seq overcomes the problems of antibodies for enrichment and sequence-motifs for evaluation (Hartstock et al., 2023). In this method first, Metabolic labeling of cells with propargyl-selenohomocysteine is performed. The labelling leads to methionine adenosyl transferase catalyzed formation of S-adenosyl-L-methionine - analogue and propargylation of methyltransferase target sites. The cells are then lysed, and poly(A) RNA is isolated and fragmented. Propargylated fragments act with biotin azide in a copper-catalyzed azide-alkyne cycloaddition and are bound to streptavidine-coated magnetic beads. Finally, on-beads reverse transcription stops at modified sites. After reverse transcription sequencing libraries are prepared and modified sites are detected as the coverage drops.

### 5.2 Non-sequencing based methods

#### 5.2.1 Radioisotope incorporation

The initial studies on RNA methylation in 1975 used the radioisotope incorporation method (Martin and Moss, 1975). First, the methyl donor, S-adenosyl-methionine is labeled with tritium, and then methyltransferase activity is measured by fluorescence as the radioactive methyl group is added onto the nucleoside (Smith et al., 1967). The limitation of this method is the unavailability of sequencing data.

#### 5.2.2 Liquid chromatography/Mass spectrometry (LC-MS)

LC-MS is regularly used to detect RNA methylation (Jora et al., 2018). A procedure of LC-MS involves nuclease P1 and alkaline phosphatase digestion of RNA. The purification of digested RNA follows them. Then ribonucleosides are separated by liquid chromatography. Then by using mass spectrometry, ribonucleoside mass chromatograms are prepared. Finally, RNA modification is quantified using the standard curve (Thüring et al., 2011). This method’s limitation is that it requires expensive instrumentation, unavailability of sequencing data and highly skilled personnel are needed.

#### 5.2.3 RT-PCR

Methylation of the 2′-hydroxyl-group of ribonucleotides (2′-O-methylation) has been noticed in various RNAs in eukaryotes. This method has used the finding that 3′-terminal RNA methylation of the 2′-hydroxyl-group of ribonucleotides 2′-O-methylation can inhibit the activity of poly(A) polymerase, an enzyme that can add the poly(A)-tail to RNA. A method by which the 2′-O-methylation level of small RNAs, such as microRNAs (miRNAs) can be directly quantified based on the poly(A)-tailed RT-qPCR technique. This method has been successfully used in *Arabidopsis thaliana* to detect the 2′-O-methylation in small RNAs. (Wang et al., 2018). In this method, first total RNA is employed for reverse transcription using stem–loop, and poly(A)-tailed primer. Based on the amplification delay, the methylation and non-methylation of small RNA is determined.

#### 5.2.4 Dot blot analysis

Dot blot analysis is a quick, easy, and cost-effective method of RNA methylation detection. The process of dot blot involves blotting RNAs directly onto a membrane substrate, then the membrane is incubated with a specific antibody (e.g., anti- m^6^A) for RNA methylation detection. Then the signals from the dot blot images can be quantified by ImageJ (Schneider et al., 2012). The result interpretation and statistical analysis of dot blot is always based on at least three biological replicates This method requires at least 20 µg of total RNA (Shen et al., 2017). For this assay, anti-m^6^A and anti-m^5^C are commercially available antibodies and mostly commonly used for this assay.The method is applicable to detect all types of RNA methylation if the specific antibodies are available.

#### 5.2.5 Immuno-northern blotting

This method has high specificity and sensitivity. In this method, first RNA is separated by gel electrophoresis. Then transferred onto a positively charged nylon membrane followed by UV cross-linking. Further incubated with the primary antibodies (e.g., m^5^C) and the secondary antibody. The specific band visualization by chemiluminescence. This method can also detect methylation in other RNAs like tRNAs, rRNAs, etc. (Mishima et al., 2015).

#### 5.2.6 Enzyme-linked Immune Sorbent Assay (ELISA)

ELISA is a simple and rapid method of RNA methylation detection. This method can be used to rapidly assess the global level of a specific RNA methylation before doing next-generation sequencing analysis. Unlike other methods, ELISA does not require denaturation, fragmentation, or electrophoresis of RNA. The process involves binding RNAs to assay well, capturing specific methylation by a primary antibody, using an enzyme-conjugated secondary antibody, and then signal detection. Different companies (e.g., EPIGENTEK, Abcam, etc.) provide kits to detect RNA methylation using ELISA.

## 6 Data analysis, software, and tools used in RNA methylation studies

RNA methylation transcriptome data analysis, i.e., epitranscriptome data analysis, varies depending on the final goal of the experiment. Epitranscriptome data generated using antibody and immunoprecipitation-based techniques follows the same principle as ChIP-Seq (Xu et al., 2020). In ChIP-Seq most commonly used tool is MACS, i.e., model-based analysis of ChIP-Seq, which follows Poisson distribution for peak calling. Similarly, MACS2 is successfully used in epitranscriptome data analysis for methylated RNA peak calling (Gaspar, 2018). MoAIMS toolkit is rapid, efficient, and easy-to-use software implemented in R. MoAIMS (Model-based analysis and inference of MeRIP-Seq) can detect enriched regions of epitranscriptome data efficiently but also evaluate the treatment effect for MeRIP-Seq treatment datasets (Zhang and Hamada, 2020). ExomePeak was one of the earliest methylation peak detection software; along with other toolkits, ExomePeak can also be used for reads mapping, RNA methylation site detection, motif discovery, differential RNA methylation analysis, and functional analysis (Meng et al., 2014). Later, RADAR and MeTDiff were developed, and both these tools have higher sensitivity and specificity than ExomePeak (Cui et al., 2018; Zhang et al., 2019). For those who do not know a computer programming language, m^6^AViewer, a graphical user interface (GUI) platform, is the best option for methylation peak analysis and visualization from sequencing data. m^6^AViewer is a novel m^6^A peak-calling algorithm that identifies high-confidence methylated residues with high precision (Antanaviciute et al., 2017). Another recently developed toolkit, m^6^A Corr, can eliminate the laboratory bias in m^6^A methylation profiles and perform profile to profile comparisons and functional analysis of hyper- (hypo-) methylated genes based on corrected methylation profiles (Li et al., 2020). The deepEA (deep epitranscriptome analysis) is another GUI toolkit with all data analysis functions like data quality check, data filtering, identification of methylation sites, functional annotation, multi-omics integrative data analysis, and prediction analysis based on machine learning. deepEA was developed based on the Galaxy framework. It can be used in windows and Linux (Copy, 2020). The information of all toolkits is summarized in **Table 2**. Finally, in the future, more tools will be generated to analyze more sophisticated RNA methylation data.

**Table 2:** Software’s/tools for RNA methylation data analysis.

Name of tool	Used in	Use	Reference
deepEA	Windows, Linux	Epitranscriptome data processing, quality check, methylation identification, functional annotation, multi-omics integrative analysis and prediction analysis based on machine learning	(Copy, 2020)
MoAIMS	R	Can detect RNA methylation enriched regions of MeRIP-Seq efficiently, also gives intuitive evaluation on treatment effect for datasets	(Zhang and Hamada, 2020)
RADAR	R	Detect differentially methylated loci in MeRIP-Seq data	(Zhang et al., 2019)
m^6^A viewer	Java, GUI	Analysis and visualization of m^6^A peaks in sequencing data	(Antanaviciute et al., 2017)
ExomePeak	R	RNA methylation site detection from MeRIP-Seq and differential analysis	(Meng et al., 2014)
MeTDiff	R	Prediction of differential m^6^A methylation sites from MeRIP-Seq data	(Cui et al., 2018)
MACS2	Linux	Originally used for Chip-Seq data analysis for peak calling, but can also be used for RNA methylated peak calling	(Gaspar, 2018)
m^6^A Corr	R	Eliminate potential laboratory bias in m^6^A methylation bias. Also performs profile-profile comparison and function analysis of hyper- (hypo) methylated genes based on correlated methylation profiles.	(Li et al., 2020)

## 7 Limitations

For most of the RNA methylation experiments, high input of total RNA is required as an abundance of m^6^A is generally less than 0.1% of the total RNA. Most researchers prefer to use mRNA for assay, but recently it has shown that miRNA, tRNA, and snRNA also undergo methylation in plants (Yu et al., 2005; Wu et al., 2021a; Parker et al., 2022). Highly proliferative cells like a callus, shoot apical meristem, and buds show a high rate of RNA methylation than non-dividing cells like leaves, roots, and flowers. So, for non-dividing cells, 100-200 µg of total RNA is required to quantify methylation accurately. For accurate quantification of mRNA methylation, several rounds of mRNA purifications are required for reliable results because mRNA constitutes 1-5% of total RNA, and other RNAs show a high rate of methylation than mRNA. Varios types of RNA methylation reported, but at each antibody-based quantification assay, only one type of RNA methylation can be studied as each antibody detects only one type of RNA methylation. There is no appropriate evidence of whether the same RNA molecules undergo cycles of methylation and demethylation (Arribas-Hernández and Brodersen, 2020).

## 8 Future perspectives

Several questions in field of plant RNA methylation remain unanswered. For example, how methylation of RNA can regulate gene expression in plants? How methylation affects the stability of cellular RNA in plants? Are reader, writer, and eraser proteins conserved among all plants? Recently, it also became possible to map RNA methylation directly by sequencing native RNAs using nanopore technologies (Begik et al., 2022). This has been applied for the detection of a RNA methylation, such as mapping of genomewide distribution of m^6^A. However, the signal modulations caused by this method is yet to be determined. Addressing these questions will significantly expand our knowledge and broaden the horizons of RNA methylation of plants.

## Author contributions

Conceptualization: HS. Writing—original draft preparation: HS, AD, and UK. Writing—review and editing: HS, AD, UK, and JH. Visualization/figures: HS, AD, and UK. Funding acquisition: UK and JH. All authors contributed to the article and approved the submitted version.

## References

[B1] Anderson S. J. Kramer M. C. Gosai S. J. Yu X. Vandivier L. E. Nelson A. D. L.. (2018). N6-methyladenosine inhibits local ribonucleolytic cleavage to stabilize mRNAs in arabidopsis. Cell Rep. 25, 1146–1157.e3. doi: 10.1016/j.celrep.2018.10.020 30380407

[B2] Antanaviciute A. Baquero-Perez B. Watson C. M. Harrison S. M. Lascelles C. Crinnion L.. (2017). M6aViewer: Software for the detection, analysis, and visualization of N6-methyladenosine peaks from m6A-seq/ME-RIP sequencing data. Rna 23, 1493–1501. doi: 10.1261/rna.058206.116 28724534 PMC5602108

[B3] Arribas-Hernández L. Brodersen P. (2020). Occurrence and functions of m6A and other covalent modifications in plant mRNA. Plant Physiol. 182, 79–96. doi: 10.1104/pp.19.01156 31748418 PMC6945878

[B4] Bartels A. Han Q. Nair P. Stacey L. Gaynier H. Mosley M.. (2018). Dynamic DNA methylation in plant growth and development. Int. J. Mol. Sci. 19, 1–17. doi: 10.3390/ijms19072144 PMC6073778 30041459

[B5] Begik O. Mattick J. S. Novoa E. M. (2022). Exploring the epitranscriptome by native RNA sequencing. RNA 28 (11), 1430–1439. doi: 10.1261/rna 36104106 PMC9745831

[B6] Bhat S. S. Bielewicz D. Gulanicz T. Bodi Z. Yu X. Anderson S. J.. (2020). mRNA adenosine methylase (MTA) deposits m6A on pri-miRNAs to modulate miRNA biogenesis in arabidopsis thaliana. Proc. Natl. Acad. Sci. U.S.A. 117, 21785–21795. doi: 10.1073/pnas.2003733117 32817553 PMC7474595

[B7] Bokar J. A. Rath-Shambaugh M. E. Ludwiczak R. Narayan P. Rottman F. (1994). Characterization and partial purification of mRNA N6-adenosine methyltransferase from HeLa cell nuclei. internal mRNA methylation requires a multisubunit complex. J. Biol. Chem. 269, 17697–17704. doi: 10.1016/S0021-9258(17)32497-3 8021282

[B8] Copy E. (2020). deepEA: A containerized web server for interactive analysis of epitranscriptome sequencing data. Plant Physiol 185 (1), 29–33. 10.1093/plphys/kiaa008 PMC8133649 33631802

[B9] Cui X. Liang Z. Shen L. Zhang Q. Bao S. Geng Y.. (2017). 5-methylcytosine RNA methylation in arabidopsis thaliana. Mol. Plant 10, 1387–1399. doi: 10.1016/j.molp.2017.09.013 28965832

[B10] Cui X. Zhang L. Meng J. Rao M. K. Chen Y. Huang Y. (2018). MeTDiff: A novel differential RNA methylation analysis for MeRIP-seq data. IEEE/ACM Trans. Comput. Biol. Bioinform. 15, 526–534. doi: 10.1109/TCBB.2015.2403355 29610101

[B11] Dai Q. Moshitch-Moshkovitz S. Han D. Kol N. Amariglio N. Rechavi G.. (2017). Nm-seq maps 2′-o-methylation sites in human mRNA with base precision. Nat. Methods 14, 695–698. doi: 10.1038/nmeth.4294 28504680 PMC5712428

[B12] David R. Burgess A. Parker B. Li J. Pulsford K. Sibbritt T.. (2017). Transcriptome-wide mapping of RNA 5-methylcytosine in arabidopsis mRNAs and noncoding RNAs. Plant Cell 29, 445–460. doi: 10.1105/tpc.16.00751 28062751 PMC5385953

[B13] Du X. Fang T. Liu Y.. (2020). Global profiling of N6-methyladenosine methylation in maize callus induction. Plant Genome 13, 1–14. doi: 10.1002/tpg2.20018 PMC12807288 33016611

[B14] Duan H. C. Wei L. H. Zhang C.. (2017). ALKBH10B is an RNA N6-methyladenosine demethylase affecting arabidopsis floral transition. Plant Cell 29, 2995–3011. doi: 10.1105/tpc.16.00912 29180595 PMC5757257

[B15] Gaspar J. M. (2018). Improved peak-calling with MACS2. bioRxiv 496521, 1–16. doi: 10.1101/496521

[B16] Greer E. L. Shi Y. (2012). Histone methylation: A dynamic mark in health, disease and inheritance. Nat. Rev. Genet. 13, 343–357. doi: 10.1038/nrg3173 22473383 PMC4073795

[B17] Hartstock K. Ovcharenko A. Kueck N. A. Spacek P. Cornelissen N. V. Huewel S.. (2023). MePMe-seq: Antibody-free simultaneous m 6 a and m 5 c mapping in mRNA by metabolic propargyl labeling and sequencing. bioRxiv. doi: 10.1101/2022.03.16.484494 PMC10630376 37935679

[B18] Hawley B. R. Jaffrey S. R. (2019). Transcriptome-wide mapping of m6A and m6Am at single-nucleotide resolution using miCLIP. Curr. Protoc. Mol. Biol. 126, 1–22. doi: 10.1002/cpmb.88 PMC6422687 30874375

[B19] Hu J. Manduzio S. Kang H. (2019a). Epitranscriptomic RNA methylation in plant development and abiotic stress responses. Front. Plant Sci. 10. doi: 10.3389/fpls.2019.00500 PMC6499213 31110512

[B20] Jora M. Lobue P. A. Ross R. L. Williams B. Addepalli B. (2018). Detection of ribonucleoside modifications by liquid chromatography coupled with mass spectrometry. Biochim Biophys Acta Gene Regul Mech. 1862 (3), 280–290. doi: 10.1016/j.bbagrm.2018.10.012 30414470 PMC6401287

[B21] Kadam U. Moeller C. A. Irudayaraj J. Schulz B. (2014a). Effect of T-DNA insertions on mRNA transcript copy numbers upstream and downstream of the insertion site in arabidopsis thaliana explored by surface enhanced raman spectroscopy. Plant Biotechnol. J. 12, 568–577. doi: 10.1111/pbi.12161 24460907

[B22] Kadam U. S. Schulz B. Irudayaraj J. M. K. (2017). Multiplex single-cell quantification of rare RNA transcripts from protoplasts in a model plant system. Plant J. 90, 1187–1195. doi: 10.1111/tpj.13537 28301688

[B23] Kadam U. S. Schulz B. Lrudayaraj J. (2014b). Detection and quantification of alternative splice sites in arabidopsis genes AtDCL2 and AtPTB2 with highly sensitive surface enhanced raman spectroscopy (SERS) and gold nanoprobes. FEBS Lett. 588, 1637–1643. doi: 10.1016/j.febslet.2014.02.061 24631541

[B24] Lemoine R. la Camera S. Atanassova R.. (2013). Source-to-sink transport of sugar and regulation by environmental factors. Front. Plant Sci. 4. 10.3389/fpls.2013.00272 PMC3721551 23898339

[B25] Li J. Huang Y. Cui Q. Zhou Y. (2020). M6Acorr: An online tool for the correction and comparison of m6a methylation profiles. BMC Bioinf. 21, 1–8. doi: 10.1186/s12859-020-3380-6 PMC6988237 31996134

[B26] Li X. Lin S. Xiang C.. (2023). CUCUME: An RNA methylation database integrating systemic mRNAs signals, GWAS and QTL genetic regulation and epigenetics in different tissues of cucurbitaceae. Comput. Struct. Biotechnol. J. 21, 837–846. doi: 10.1016/j.csbj.2023.01.012 36698975 PMC9842799

[B27] Li Y. Wang X. Li C.. (2014). Transcriptome-wide N6-methyladenosine profiling of rice callus and leaf reveals the presence of tissue-specific competitors involved in selective mRNA modification. RNA Biol. 11, 1180–1188. doi: 10.4161/rna.36281 25483034 PMC5155352

[B28] Liang Z. Riaz A. Chachar S.. (2020). Epigenetic modifications of mRNA and DNA in plants. Mol. Plant 13, 14–30. 31863849 10.1016/j.molp.2019.12.007

[B29] Lim W. A. Pawson T. (2010). Phosphotyrosine signaling: Evolving a new cellular communication system. Cell 142, 661–667. doi: 10.1016/j.cell.2010.08.023 20813250 PMC2950826

[B30] Linder B. Grozhik A. V. Olarerin-George A. O. Meydan C. Mason C. E. Jaffrey S. R. (2015). Single-nucleotide-resolution mapping of m6A and m6Am throughout the transcriptome. Nat. Methods 12, 767–772. doi: 10.1038/nmeth.3453 26121403 PMC4487409

[B31] Liu C. Lu F. Cui X. Cao X. (2010). Histone methylation in higher plants. Annu. Rev. Plant Biol. 61, 395–420. doi: 10.1146/annurev.arplant.043008.091939 20192747

[B32] Liu G. Wang J. Hou X. (2020). Transcriptome-wide N6-methyladenosine (M6a) methylome profiling of heat stress in pak-choi (brassica rapa ssp. chinensis). Plants 9, 1–12. doi: 10.3390/plants9091080 PMC7570095 32842619

[B33] Lucas W. J. Groover A. Lichtenberger R. Furuta K. Yadav S.-R. Helariutta Y.. (2013). The plant vascular system: Evolution, development and functions. J. Integr. Plant Biol. 55, 294–388. doi: 10.1111/jipb.12041 23462277

[B34] Luo J. H. Wang Y. Wang M. Zhang L.Y. Peng H. R. Zhou Y.Y.. (2020). Natural variation in RNA m6a methylation and its relationship with translational status. Plant Physiol. 182, 332–344. doi: 10.1104/pp.19.00987 31591151 PMC6945879

[B35] Manduzio S. Kang H. (2021). RNA Methylation in chloroplasts or mitochondria in plants. RNA Biol. 18, 2127–2135. doi: 10.1080/15476286.2021.1909321 33779501 PMC8632092

[B36] Martin S. A. Moss B. (1975). Modification of RNA by mRNA guanylyltransferase and mRNA (guanine 7) methyltransferase from vaccinia virions. J. Biol. Chem. 250, 9330–9335. doi: 10.1016/s0021-9258(19)40647-9 1194287

[B37] Martínez-Pérez M. Aparicio F. López-Gresa M. P. Bellés María J. Sánchez-Navarro J.A. Pallás V. (2017). Arabidopsis m6A demethylase activity modulates viral infection of a plant virus and the m6A abundance in its genomic RNAs. Proc. Natl. Acad. Sci. U.S.A. 114, 10755–10760. doi: 10.1073/pnas.1703139114 28923956 PMC5635872

[B38] Meng J. Lu Z. Liu H. Zhang L. Zhang S. Chen Y.. (2014). A protocol for RNA methylation differential analysis with MeRIP-seq data and exomePeak R/Bioconductor package. Methods 69, 274–281. doi: 10.1016/j.ymeth.2014.06.008 24979058 PMC4194139

[B39] Meyer K. D. Saletore Y. Zumbo P. Elemento O. Mason C. E. Jaffrey S. R. (2012). Comprehensive analysis of mRNA methylation reveals enrichment in 3′ UTRs and near stop codons. Cell 149, 1635–1646. doi: 10.1016/j.cell.2012.05.003 22608085 PMC3383396

[B40] Mishima E. Jinno D. Akiyama Y. Itoh K. Nankumo S. Shima H.. (2015). Immuno-northern blotting: Detection of RNA modifications by using antibodies against modified nucleosides. PloS One 10, 1–17. doi: 10.1371/journal.pone.0143756 PMC4659547 26606401

[B41] Mongan N. P. Emes R. D. Archer N. (2019). Detection and analysis of RNA methylation. F1000Res 8, 1–12. doi: 10.12688/f1000research.17956.1 PMC6489984 31069058

[B42] Nichols J. L. (1980). N6-methyladenosine in maize poly(A)-containing RNA. Plant Sci. Lett. 15, 357–361. doi: 10.1016/0304-4211(79)90141-X

[B43] Nucleosides I. Chromatography T. (1980). Post - transcriptional modifications of oat coleoptile ribonucleic acids. Eur. J. Biochem. 277, 271–277. 10.1111/j.1432-1033.1980.tb04425.x 6154573

[B44] Parker M. T. Knop K. Sherwood A. V. Schurch N. J. Mackinnon K. Gould P. D.. (2019). Nanopore direct RNA sequencing maps an arabidopsis N6 methyladenosine epitranscriptome. bioRxiv. doi: 10.1101/706002 PMC6959997 31931956

[B45] Parker M. T. Soanes B. K. Kusakina J. Larrieu A. Knop K. Breidenbach N. J. F.. (2022). m6A modification of U6 snRNA modulates usage of two major classes of pre-mRNA 5’ splice site. Elife 11. doi: 10.7554/eLife.78808 PMC9803359 36409063

[B46] Pastore C. Topalidou I. Forouhar F. Yan A. C. Levy M. Hun J. F. (2012). Crystal structure and RNA binding properties of the RNA recognition motif (RRM) and AlkB domains in human AlkB homolog 8 (ABH8), an enzyme catalyzing tRNA hypermodification. J. Biol. Chem. 287, 2130–2143. doi: 10.1074/jbc.M111.286187 22065580 PMC3265892

[B47] Ruocco M. Ambrosino L. Jahnke M. Chiusano M. L. Barrote I. Procaccini G.. (2020). M6A RNA methylation in marine plants: First insights and relevance for biological rhythms. Int. J. Mol. Sci. 21, 1–21. doi: 10.3390/ijms21207508 PMC7589960 33053767

[B48] Růžička K. Zhang M. Campilho A. Bodi Z. Kashif M. Saleh M.. (2017). Identification of factors required for m6A mRNA methylation in arabidopsis reveals a role for the conserved E3 ubiquitin ligase HAKAI. New Phytol. 215, 157–172. doi: 10.1111/nph.14586 28503769 PMC5488176

[B49] Schaefer M. Pollex T. Hanna K. Lyko F. (2009). RNA Cytosine methylation analysis by bisulfite sequencing. Nucleic Acids Res. 37. doi: 10.1093/nar/gkn954 PMC2632927 19059995

[B50] Schneider C. A. Rasband W. S. Eliceiri K. W. (2012). NIH Image to ImageJ: 25 years of image analysis. Nat. Methods 9, 671–675. 22930834 10.1038/nmeth.2089 PMC5554542

[B51] Shen L. Liang Z. Gu X. Chen Y. Teo Z.W.N. Hou X.. (2016). N6-methyladenosine RNA modification regulates shoot stem cell fate in arabidopsis. Dev. Cell 38, 186–200. doi: 10.1016/j.devcel.2016.06.008 27396363 PMC6364302

[B52] Shen L. Liang Z. Yu H. (2017). Dot blot analysis of N6-methyladenosine RNA modification levels. Bio Protoc. 7, 4–8. doi: 10.21769/bioprotoc.2095 PMC8376565 34458425

[B53] Shi Y. Wang H. Wang J. Liu X. Lin F. Lu J. (2019). N6-methyladenosine RNA methylation is involved in virulence of the rice blast fungus pyricularia oryzae (syn. Magnaporthe oryzae). FEMS Microbiol. Lett. 366, 1–10. doi: 10.1093/femsle/fny286 30535195

[B54] Shoaib Y. Usman B. Kang H. Jung K. H. (2022). Epitranscriptomics: An additional regulatory layer in plants’ development and stress response. Plants 11. 10.3390/plants11081033 PMC9027318 35448761

[B55] Smith K. D. Armstrong J. L. McCarthy B. J. (1967). THE INTRODUCTION OF RADIOISOTOPES INTO RNA BY METHYLATION IN VITRO. Biochim. Biophys. Acta 142. 10.1016/0005-2787(67)90615-6 4861434

[B56] Suzuki M. M. Bird A. (2008). DNA Methylation landscapes: Provocative insights from epigenomics. Nat. Rev. Genet. 9, 465–476. doi: 10.1038/nrg2341 18463664

[B57] Thüring K. Schmid K. Keller P. Helm M. (2011). LC-MS analysis of methylated RNA. RNA Methylation: Methods Protocols Methods Mol. Biol., 2–6. 10.1007/978-1-4939-6807-7_1 28349450

[B58] Tieu Ngoc L. N. Jung Park S. Thi Huong T. Lee Ho K. Kang H. (2021). N4-methylcytidine ribosomal RNA methylation in chloroplasts is crucial for chloroplast function, development, and abscisic acid response in arabidopsis. J. Integr. Plant Biol. 63, 570–582. doi: 10.1111/jipb.13009 32876986

[B59] Vandivier L. E. Campos R. Kuksa P. P. Silverman I. M. Wang L.-S. Gregory B. D. (2015). Chemical modifications mark alternatively spliced and uncapped messenger RNAs in arabidopsis. Plant Cell 27, 3024–3037. doi: 10.1105/tpc.15.00591 26561561 PMC4682304

[B60] Wang T. Li X. Zhang X. Wang Q. Liu W. Lu X.. (2021a). RNA Motifs and modification involve in RNA long-distance transport in plants. Front. Cell Dev. Biol. 9. doi: 10.3389/fcell.2021.651278 PMC8047152 33869208

[B61] Wang N. Qu S. Sun W. Zeng Z. Liang H. Zhang C. Y.. (2018). Direct quantification of 3 ′ terminal 2 ′ -o-methylation of small RNAs by RT-qPCR. Rna 24, 1520–1529. doi: 10.1261/rna.065144.117 30076204 PMC6191718

[B62] Wang L. Zhuang H. Fan W. Zhang X. Dong H. Yang H.. (2022). m6A RNA methylation impairs gene expression variability and reproductive thermotolerance in arabidopsis. Genome Biol. 23, 244. doi: 10.1186/s13059-022-02814-8 36419179 PMC9686071

[B63] Wei L. H. Song P. Wang Y. Lu Z. Tang Q. Yu Q.. (2018). The m6A reader ECT2 controls trichome morphology by affecting mRNA stability in arabidopsis. Plant Cell 30, 968–985. doi: 10.1105/tpc.17.00934 29716990 PMC6002187

[B64] Wong C. E. Zhang S. Xu T. Zhang Y. Teo Z. W. N. Yan A.. (2023). Shaping the landscape of *N6* -methyladenosine RNA methylation in arabidopsis. Plant Physiol. doi: 10.1093/plphys/kiad010 PMC10022626 36627133

[B65] Wu S. Wang Y. Wang J. Li X. Li J. Ye K. (2021a). Profiling of RNA ribose methylation in arabidopsis thaliana. Nucleic Acids Res. 49, 4104–4119. doi: 10.1093/nar/gkab196 33784398 PMC8053127

[B66] Xu X. Wei X. Xie H. (2020). Advances in methods and software for RNA cytosine methylation analysis. Genomics 112, 1840–1846. doi: 10.1016/j.ygeno.2019.10.017 31678155 PMC7195603

[B67] Yang Y. Hsu P. J. Chen Y. S. Yang Y. G. (2018). Dynamic transcriptomic m6A decoration: Writers, erasers, readers and functions in RNA metabolism. Cell Res. 28, 616–624. doi: 10.1038/s41422-018-0040-8 29789545 PMC5993786

[B68] Yang L. Perrera V. Saplaoura E. Apelt F. Bahin M. Kramdi A.. (2019a). m5C methylation guides systemic transport of messenger RNA over graft junctions in plants. Curr. Biol. 29, 2465–2476.e5. doi: 10.1016/j.cub.2019.06.042 31327714

[B69] Young Hee Choi A.-M. Y. (2014). Unique features of the m6A methylome in arabidopsis thaliana. Physiol. Behav. 176, 139–148. doi: 10.1038/ncomms6630.Unique PMC4248235 25430002

[B70] Yu B. Yang Z. Li J. Minakhina S. Yang M. Padgett R.W.. (2005). Methylation as a crucial step in plant microRNA biogenesis. Science 307, 932–935. doi: 10.1126/science.1107130 15705854 PMC5137370

[B71] Zaccara S. Ries R. J. Jaffrey S. R. (2019). Reading, writing and erasing mRNA methylation. Nat. Rev. Mol. Cell Biol. 20, 608–624. doi: 10.1038/s41580-019-0168-5 31520073

[B72] Zhang Y. Hamada M. (2020). MoAIMS: Efficient software for detection of enriched regions of MeRIP-seq. BMC Bioinf. 21, 1–12. doi: 10.1186/s12859-020-3430-0 PMC7071693 32171255

[B73] Zhang Z. Zhan Q. Eckert M.. (2019). RADAR: Differential analysis of MeRIP-seq data with a random effect model. Genome Biol. 20, 1–17. doi: 10.1186/s13059-019-1915-9 31870409 PMC6927177

[B74] Zheng H. Li S. Zhang X. Sui N. (2020). Functional implications of active N6-methyladenosine in plants. Front. Cell Dev. Biol. 8. doi: 10.3389/fcell.2020.00291 PMC7202093 32411708

[B75] Zhong S. Li H. Bodi Z. Button J. Vespa L. Herzog M.. (2008). MTA is an arabidopsis messenger RNA adenosine methylase and interacts with a homolog of a sex-specific splicing factor. Plant Cell 20, 1278–1288. doi: 10.1105/tpc.108.058883 18505803 PMC2438467

[B76] Zhou L. Tian S. Qin G. (2019). RNA Methylomes reveal the m6A-mediated regulation of DNA demethylase gene SlDML2 in tomato fruit ripening. Genome Biol. 20, 1–23. doi: 10.1186/s13059-019-1771-7 31387610 PMC6683476

[B77] Zou M. Mu Y. Chai X. Ouyang M. Yu L. J. Zhang L.. (2020). The critical function of the plastid rRNA methyltransferase, CMAL, in ribosome biogenesis and plant development. Nucleic Acids Res. 48, 3195–3210. doi: 10.1093/nar/gkaa129 32095829 PMC7102989

